# Study on the Immobilization of Horseradish Peroxidase on a Multi-Level Composite Carrier SiO_2_@MnO_2_@MAF-7

**DOI:** 10.3390/ma19020254

**Published:** 2026-01-08

**Authors:** Mengjie Huang, Baihui Zhang, Xiangyu Jiang, Maojie Jiang, Peng Yin, Xuan Fang, Yanna Lin, Fuqiang Ma

**Affiliations:** 1 Shandong Laboratory of Advanced Biomaterials and Medical Devices in Weihai, Weihai 264200, Chinazbh16168@163.com (B.Z.); z21140051@s.upc.edu.cn (X.J.); 15206868591@163.com (X.F.); 2 Medical Enzyme Engineering Center, CAS Key Laboratory of Bio-Medical Diagnostics, Suzhou Institute of Biomedical Engineering and Technology, Chinese Academy of Sciences, Suzhou 215163, China

**Keywords:** SiO_2_@MnO_2_@MAF-7, immobilization, multi-level structure, stability

## Abstract

**Highlights:**

**What are the main findings?**

- Hierarchical Immobilization Superiority: The SiO_2_@MnO_2_@MAF-7 carrier immobilized HRP via three strategies (adsorption/cross-linking/encapsulation), with MAF-7 encapsulation showing the highest efficiency (85.03%) and 42% lower Km, indicating enhanced substrate affinity.
- Synergistic Stability Enhancement: MnO_2_’s peroxidase-like activity combined with MAF-7’s confinement effect improved HRP’s activity retention by 35–50% at 80 °C/pH 4–9, maintaining 65% activity after five reuse cycles.
- Structure-Mechanism Correlation: BET/XRD confirmed MAF-7’s mesopores (12.47 nm) matched HRP size, while FTIR/docking revealed HRP-MAF-7 stabilization via π-π stacking and H-bonds, clarifying the molecular-level protection mechanism.

**What are the implications of the main findings?**

- Industrial Potential: The composite carrier addresses free HRP’s instability and unrecoverability, offering a scalable solution for oxidoreductase applications.
- Generalizable Design: The “rigid core-catalytic shell-porous framework” strategy can be extended to other enzymes (e.g., laccase or glucose oxidase).
- Mechanistic Insight: The revealed Mn^2+^/Zn^2+^ dual-metal coordination with HRP guides future designs of bioinspired catalysts and stimuli-responsive carriers.

**Abstract:**

This study addresses the issues of poor stability and difficulty in recovery of free horseradish peroxidase (HRP) by developing a multi-level composite immobilized carrier that combines high loading capacity with long-term stability. The SiO_2_@MnO_2_@MAF-7 core–shell structured carrier was prepared via a solvothermal self-assembly method. Three immobilization strategies—adsorption, covalent cross-linking, and encapsulation—were systematically compared for their immobilization efficacy on HRP. The material structure was analyzed using techniques such as specific surface area analysis (BET), X-ray diffraction (XRD), and Fourier transform infrared spectroscopy (FTIR) to characterize the material structure. Enzyme kinetic parameter determination experiments were conducted to systematically evaluate the performance advantages of the immobilized enzyme. BET analysis showed that SiO_2_@MnO_2_@MAF-7 had a specific surface area of 251.99 m^2^/g and a mesoporous area of 12.47 nm, and its HRP loading was 50.37 U/mg (immobilization efficiency 85.03%). Compared with free HRP, the Km value of the immobilized enzyme was decreased by 42%, the activity retention rate was increased by 35–50% at 80 °C and pH 4–9, and the activity was maintained by 65% after five repeated uses. In this study, MAF-7 was combined with MnO_2_/SiO_2_ for HRP immobilization for the first time, and the triple effect of rigid support-catalytic synergy-confined protection synergistically improved the stability of the enzyme, providing a new strategy for the industrial application of oxidoreductases.

## 1. Introduction

As a highly efficient biocatalyst, horseradish peroxidase (HRP) has attracted much attention in the fields of biosensing [1,2,3,4] and biomanufacturing [5,6]. However, the use of free HRP is severely restricted in industrial applications due to issues such as easy inactivation, difficulty in recovery, and limitations in operational conditions [7]. In recent years, immobilization technologies based on nanomaterials [2] and porous frameworks [8,9] have become important strategies to address the aforementioned challenges. However, single material systems often struggle to achieve high loading capacity, retention of activity, and long-term stability simultaneously due to functional limitations [10], including mass transfer resistance effects, reduction in effective loading sites caused by the agglomeration of nanoparticles [11], and shielding effects on enzyme active sites and decreased environmental tolerance induced by strong covalent modifications [12]. In response to this issue, the construction of multi-level composite carriers to synergistically integrate the advantageous characteristics of different materials has become a frontier direction in the research of immobilized enzymes [13,14]. For example, the ZIF-8-coated magnetic Fe_3_O_4_@SiO_2_ carrier can simultaneously improve the magnetic recovery efficiency and acid stability of immobilized enzymes [15]; modifying mesoporous carbon loaded with horseradish peroxidase (HRP) with MnO_2_ nanosheets can significantly enhance electrochemical sensing responses [16]. However, there have been no systematic research reports to date on the composite of MAF-7 crystal materials with metal oxides to construct novel enzyme immobilization carriers. The interfacial synergy mechanisms between the layers, the regulatory patterns of enzyme conformation, and the mechanisms of catalytic enhancement still require in-depth exploration. Moreover, the controllable preparation of composite materials and the challenges of batch reproducibility also face significant obstacles.

This study proposes a novel strategy for the immobilization of horseradish peroxidase (HRP) using a multi-level composite material based on SiO_2_@MnO_2_@MAF-7. In this composite, the core of nanosilica (SiO_2_) serves as a rigid supporting matrix, providing high mechanical stability and abundant surface hydroxyl groups [17]; the intermediate layer of manganese dioxide (MnO_2_) not only exhibits a significant synergistic catalytic effect [18], but its peroxidase-like activity can also optimize the spatial orientation of enzyme molecules through a metal coordination mechanism [19]. This coordination effect can induce the hematin groups to form a directional arrangement at the reaction interface, thereby significantly enhancing the utilization of active sites. The outer layer, the metal–organic framework MAF-7, enables high-density loading of enzyme molecules due to its highly ordered mesoporous structure, and the three-dimensional pore structure effectively suppresses the deactivation phenomenon caused by enzyme aggregation through spatial confinement effects [20]. Moreover, the organic ligands of MAF-7 can directionally capture and protect HRP molecules through electrostatic interactions or covalent modifications, optimizing the exposure of active sites and reducing mass transfer resistance [21,22]. This three-layer composite structure achieves synergistic optimization of material performance through a functionally graded design: the SiO_2_ core layer imparts basic mechanical strength to the support, the MnO_2_ interlayer provides interface activation functions, and the epitaxially grown MAF-7 porous framework effectively regulates mass transfer kinetics and enhances enzyme-support interfacial interactions due to its molecular sieve effect and coordination characteristics, thereby overcoming the performance bottlenecks of traditional single materials. The SiO_2_@MnO_2_@MAF-7 composite material demonstrates outstanding potential for enzyme immobilization due to its unique “core-shell-frame” hierarchical structure, providing a multifunctional platform for the efficient immobilization of horseradish peroxidase (HRP) and offering theoretical guidance for the rational design of hierarchical composite supports in the field of biocatalysis, with the potential to promote the advancement of enzyme immobilization technology towards greater intelligence and functional integration.

## 2. Materials and Methods

### 2.1. Chemicals and Reagents

Tetraethyl orthosilicate, (3-aminopropyl)triethoxysilane (APTES), 4-chloromethylphenylboronic acid (4-CMPBA), and 3,3′,5,5′-tetramethylbenzidine (TMB) were purchased from MCL Biomedical Co., Ltd. (Shanghai, China). N-[3-methylsilylpropyl]ethylenediamine and ethyl acetate were obtained from Aladdin Biochemical Technology Co., Ltd. (Shanghai, China). Horseradish peroxidase (HRP), Coomassie Brilliant Blue G-250, glutaraldehyde (GA), polyvinylpyrrolidone (PVP), glutamic acid, 3-methyl-1,2,4-triazole, Zn(NO_3_)_2_·6H_2_O, MnSO_4_, and (NH_4_)_2_S_2_O_8_ were sourced from Merck Biochemical Technology Co., Ltd. (Shanghai, China). Ethanol was acquired from Tianjin Zhiyuan Chemical Reagent Co., Ltd. (Tianjin, China). NaOH was obtained from Tianjin Damao Chemical Reagent Factory (Tianjin, China). Hexadecyltrimethylammonium bromide (CTAB) was purchased from Tianjin Guangfu Science and Technology Development Co., Ltd. (Tianjin, China). Phosphate-buffered saline (PBS 100 mM) was obtained from Sangnong Biotech Co., Ltd. (Shanghai, China), and NH_3_·H_2_O was sourced from Yantai Far East Fine Chemical Co., Ltd. (Yantai, Shandong Province, China).

### 2.2. Apparatus

The absorbance of the oxidized TMB generated after the enzyme-catalyzed reaction was measured at 652 nm using a UV-Vis spectrophotometer (Shimadzu UV-2600i, Shimadzu Management (China) Co., Ltd., Shanghai, China). The morphology of the material was characterized using SEM (Zeiss Sigma 360, Carl Zeiss (Shanghai) Management Co., Ltd., Shanghai, China) and TEM (FEI Talos F200S, Thermo Fisher Scientific (China) Co., Ltd., Shanghai, China). XRD (Bruker D8 Advance, Brook (Beijing) Technology Co., Ltd., Beijing, China) was used to characterize the crystal structure of the material. BET (Micromeritics ASAP 2460, McMurray-Tec (Shanghai) Instrument Co., Ltd., Shanghai, China) was used to obtain information on the specific surface area and pore size of the material (N_2_ adsorption–desorption testing was conducted at 77 K using a Micromeritics ASAP 2460 instrument. Samples were degassed at 120 °C for 12 h prior to measurement. Isotherms were classified according to IUPAC standards [23]. BET surface area was calculated using the Brunauer–Emmett–Teller theory (P/P_0_ = 0.05–0.35). Pore size distribution was derived from the Barrett–Joyner–Halenda (BJH) model applied to the desorption branch of isotherms. The surface functional groups of the material were characterized using FTIR (Thermo Fisher Scientific Nicolet iS20, Thermo Fisher Scientific (China) Co., Ltd., Shanghai, China).

### 2.3. Methods of Enzyme Immobilization and Loading Capacity

#### 2.3.1. Synthesis of SiO_2_@MnO_2_@mSiO_2_

1. Preparation of spherical SiO_2_;

Measure 10 mL of ethanol, 1 mL of deionized water, 2 mL of NH_3_·H_2_O, and 500 μL of tetraethyl orthosilicate, and mix them evenly in a 25 mL beaker. Stir at room temperature for 12 h. Upon completion of the reaction, centrifuge to collect the product, wash it three times each with ethanol and deionized water, and store it by freeze-drying.

2. Preparation of SiO_2_@MnO_2_;

Weigh 50 mg of SiO_2_, 0.68 mg of MnSO_4_, and 0.92 g of (NH_4_)_2_S_2_O_8_, and dissolve them in 40 mL of deionized water. Ultrasonically disperse until uniform, then transfer the mixed solution to a high-pressure reaction vessel and react at 120 °C for 2 h. After cooling to room temperature, centrifuge to collect the product, and wash it three times each with ethanol and deionized water, then freeze-dry for storage.

3. Preparation of SiO_2_@MnO_2_@mSiO_2_

Add 300 μL of NaOH (0.2 M) and 45 mL of CTAB (2.2 mg/mL) to a 100 mL round-bottom flask, and sonicate to achieve uniform dispersion. Heat and stir, and when the temperature reaches 70 °C, add 750 μL of SiO_2_@MnO_2_ (3.15 mg/mL) every 5 min, totaling three additions, ensuring thorough mixing. Under stirring conditions, sequentially add a mixture of 500 μL of N-[3-trimethylsilylpropyl]-ethylenediamine and tetraethyl orthosilicate (in a volume ratio of 1:4), 50 μL of APTES, and 3 mL of ethyl acetate to the system. After a reaction time of 15 min, add 50 μL of APTES every 10 min for a total of three additions. After the mixture is refluxed for 3 h, centrifuge to collect the product, wash with ethanol, and then redisperse in 40 mL of ethanol. Reflux overnight at 80 °C to completely remove CTAB, then centrifuge again, wash with ethanol, and dry.

#### 2.3.2. Direct Adsorption Method for Fixing HRP

Add 2 mg of SiO_2_@MnO_2_@mSiO_2_ to 1 mL of deionized water and disperse uniformly using ultrasonic treatment. Dissolve HRP in PBS with a pH of 7.4 to prepare a concentration of 1 mg/mL HRP. Mix uniformly in volume ratios of materials to HRP of 10:1, 10:2, 10:3, 10:4, 10:5, 10:6, 10:7, 10:8, and 10:9, then add PBS to make up to equal volume and oscillate at room temperature for 30 min. After centrifugation, collect the supernatant of the immobilized enzyme and measure the concentration of HRP using the Coomassie Brilliant Blue method. The absorbance is measured at 595 nm using a UV-Vis spectrophotometer, averaging three parallel measurements for each group.

The loading capacity is calculated using the following formula.

HRP_loading_ = (C_0_ − C_e_) × V/m,

C_0_ is the concentration of HRP before adsorption, mg/mL; C_e_ is the concentration of HRP when adsorption reaches equilibrium, mg/mL; V is the total volume of the reaction system, mL; m is the amount of SiO_2_@MnO_2_@mSiO_2_ added, mg.

#### 2.3.3. Cross-Linking Method for Fixing HRP

This article selected commonly used covalent cross-linking agent GA and affinity cross-linking agent 4-CMPBA for enzyme immobilization. The aldehyde groups at both ends of GA form covalent bonds with the carrier and the amino groups of lysine on the surface of HRP through a Schiff base reaction, achieving HRP immobilization; 4-CMPBA can specifically bind to the glycosyl group of HRP through its boric acid group (-B(OH)_2_), while its chloromethyl group (-CH_2_Cl) can also covalently interact with the amino or sulfhydryl groups on the surface of HRP, thereby facilitating HRP immobilization.

1. Using GA as a cross-linking agent

Mix SiO_2_@MnO_2_@mSiO_2_ (2 mg/mL) and GA (0.5~5%) thoroughly, incubate at 37 °C for 2 h. After the reaction is complete, centrifuge to remove the supernatant, wash repeatedly with PBS, and then re-disperse in PBS. Add HRP (1 mg/mL) and mix uniformly, allowing it to react overnight at 4 °C. After the reaction, determine the loading capacity using the aforementioned method.

2. Using 4-CMPBA as a cross-linking agent

Dissolve 4-CMPBA in DMSO and prepare a 0.01 mM to 0.15 mM solution of 4-CMPBA by adding deionized water. Mix 4-CMPBA with SiO_2_@MnO_2_@mSiO_2_ (3.3 mg/mL) uniformly and stir at 65 °C for 2 h. After the reaction is complete, collect the product to obtain SiO_2_@MnO_2_@mSiO_2_-4-CMPBA. Mix SiO_2_@MnO_2_@mSiO_2_-4-CMPBA (2 mg/mL) with HRP (1 mg/mL) uniformly and stir at 20 °C for 1 h. After the reaction is complete, detect the enzyme loading according to the aforementioned method.

#### 2.3.4. SiO_2_@MnO_2_-HRP@MAF-7 Embedding Method for Immobilizing HRP

In a beaker, while stirring, sequentially add SiO_2_@MnO_2_ (1 mg/mL), PVP (50 mg/mL), glutamic acid (9.1 mg/mL), 3-methyl-1,2,4-triazole (800 mM), NH_3_·H_2_O (10%), and Zn(NO_3_)_2_·6H_2_O (40 mM). Mix uniformly and stir for 30 min to allow the MAF-7 to uniformly grow on the surface of SiO_2_@MnO_2_. Subsequently, add HRP to the reaction system and stir overnight at 4 °C. After the reaction, measure the enzyme loading as described above.

### 2.4. Enzyme Activity Test

The TMB colorimetric method is employed to detect the activity of immobilized enzymes and free HRP. Under the catalysis of HRP, TMB is oxidized to generate a blue product, which has a maximum absorption peak at 652 nm. TMB is added to a solution containing HRP and H_2_O_2_ and mixed rapidly to ensure uniformity, followed by immediate measurement of the absorbance at a wavelength of 652 nm using a UV–visible spectrophotometer. The collected UV/visible absorbance data are fitted to the Michaelis–Menten equation to calculate Km, which is used to determine the catalytic activity of free enzymes and immobilized enzymes.

### 2.5. The Interaction Between HRP and SiO_2_@MnO_2_@MAF-7

To investigate the binding energy and interaction mode between HRP and SiO_2_@MnO_2_@MAF-7, we employed Autodock Vina 1.2.2 software to perform molecular docking between HRP and 3-methyl-1,2,4-triazole [24]. The molecular structure of 3-methyl-1,2,4-triazole was obtained from the PubChem compound database (https://pubchem.ncbi.nlm.nih.gov/ (16 May 2025)). The 3D coordinates of HRP were downloaded from the PDB. We first prepared the protein and ligand files, converting all protein and molecular files to PDBQT format, removing all water molecules, and adding polar hydrogen atoms. The grid box was centered to cover the structural domain of each protein and accommodate free molecular movement. The interface pocket was set as a square pocket of 30 Å × 30 Å × 30 Å, with a grid point distance of 0.05 nm. The molecular docking study was conducted using Autodock Vina 1.2.2 (http://autodock.scripps.edu/ (19 May 2025) for model visualization.

### 2.6. Stability and Reusability of Free HRP and SiO_2_@MnO_2_-HRP@MAF-7

1. Stability of pH Value

Add free HRP and SiO_2_@MnO_2_-HRP@MAF-7 to 1 mL of buffer solution with a pH range of 2.0 to 12.0, and after incubating at room temperature for 0.5 to 5 h, assess the residual activity of HRP. The buffer solution used in the experiment is PBS (100 mM).

2. Thermal Stability

Incubate the free HRP and SiO_2_@MnO_2_-HRP@MAF-7 at different temperatures (40, 80 °C, pH = 7.4) for 0.5 to 3 h. After cooling the samples to room temperature, measure the enzyme activity. The data corresponding to the highest activity is considered as 100%, and similarly for the other measurements.

3. Storage Stability

Prepare the solution samples and the powder samples of free HRP and SiO_2_@MnO_2_-HRP@MAF-7, storing the samples at room temperature and at 4 °C for 56 days, measuring the enzyme activity every 7 days.

4. Reusability

Add TMB and H_2_O_2_ to the SiO_2_@MnO_2_-HRP@MAF-7 solution for testing. After the reaction was completed, centrifuge and wash the sample, followed by the addition of TMB and H_2_O_2_ again as substrates for testing. Measure the residual enzyme activity after each cycle, and collect the supernatant by centrifugation after each cycle. Confirm the amount of HRP leakage by measuring the absorbance of the supernatant at 595 nm (using the Coomassie Brilliant Blue method). The initial enzyme activity was designated as 100%.

All of the aforementioned HRP activity measurements were performed in triplicate, and the average values were obtained.

## 3. Results and Discussion

### 3.1. Characterization of SiO_2_, SiO_2_@MnO_2_, SiO_2_@MnO_2_@mSiO_2_, and SiO_2_@MnO_2_@MAF-7

**Figure 1:**
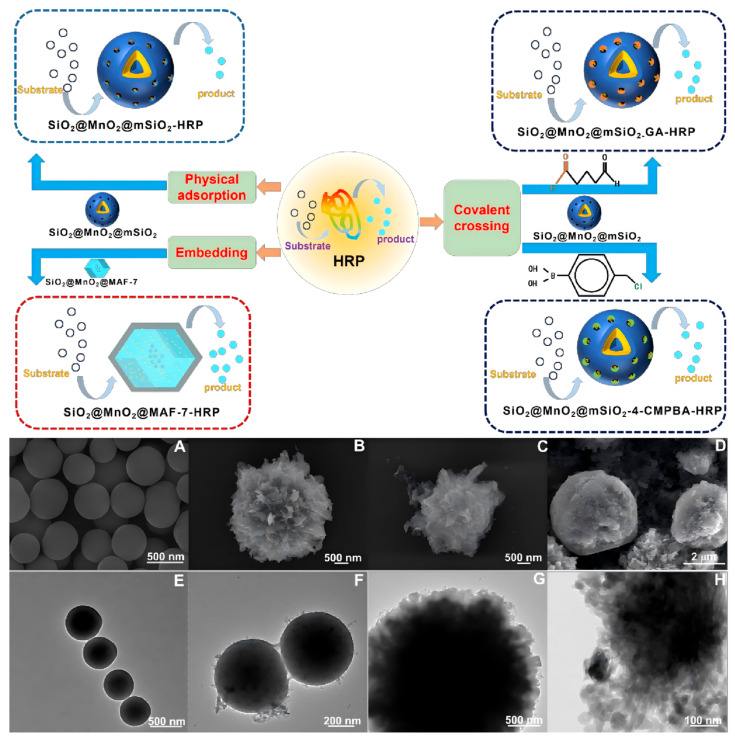
Schematic comparison of four HRP immobilization strategies (direct adsorption, GA cross-linking, 4-CMPBA cross-linking, and MAF-7 encapsulation). SEM and TEM images of SiO_2_ (**A**,**E**), SiO_2_@MnO_2_ (**B**,**F**), SiO_2_@MnO_2_@mSiO_2_ (**C**,**G**), and SiO_2_@MnO_2_@MAF-7 (**D**,**H**).

The SEM and TEM characterizations of SiO_2_, SiO_2_@MnO_2_, SiO_2_@MnO_2_@mSiO_2_, and SiO_2_@MnO_2_@MAF-7 are shown in Figure 1. The SEM (Figure 1A) and TEM (Figure 1E) images of SiO_2_ indicate that the surface of SiO_2_ is smooth and spherical. After encapsulating with MnO_2_, the surface of SiO_2_@MnO_2_ (Figure 1B,F) becomes rougher, exhibiting a flake-like MnO_2_ structure. Figure 1C,G show that the surface shell thickness of SiO_2_@MnO_2_@mSiO_2_ further increases, and after encapsulating with MAF-7, the surface of SiO_2_@MnO_2_@MAF-7 (Figure 1D,H) becomes smooth while presenting a new polyhedral crystal structure, providing preliminary evidence for the successful synthesis of SiO_2_@MnO_2_@MAF-7.

**Figure 2:**
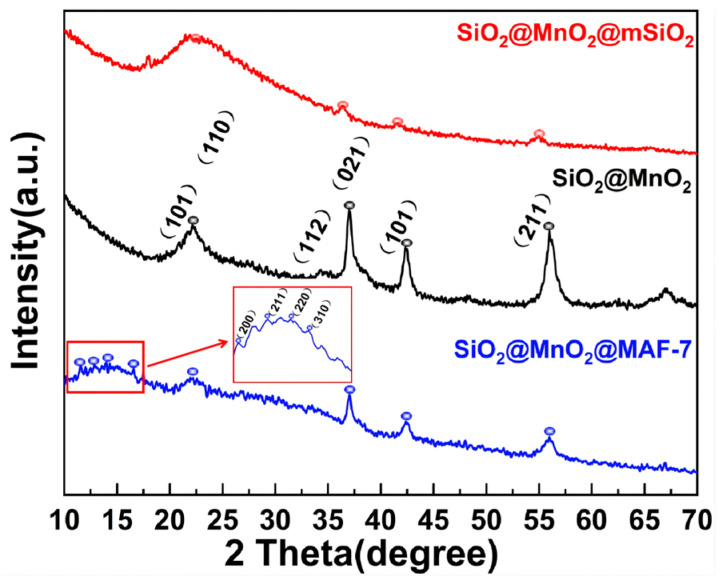
XRD images of SiO_2_@MnO_2_, SiO_2_@MnO_2_@mSiO_2_, and SiO_2_@MnO_2_@MAF-7.

The XRD spectra of the material are shown in Figure 2. The diffraction peaks of SiO_2_@MnO_2_ exhibit characteristic peaks for both SiO_2_ and MnO_2_. The peak at 22.15° corresponds to the (101) crystal plane of SiO_2_ and the (110) crystal plane of MnO_2_ [25,26], indicating that this intermediate contains two crystal structures. The (112) crystal plane of SiO_2_ and the (021) crystal plane of MnO_2_ coincide at 37°, while 42.37° and 55.97° correspond to the (101) and (211) crystal planes of MnO_2_, respectively, which align with the main peaks in the MnO_2_ standard card [26]. The spectra of SiO_2_@MnO_2_@mSiO_2_ and SiO_2_@MnO_2_ show good overlap, with identical diffraction peaks appearing at 2θ = 21.87°, 36.45°, 41.63°, and 54.99°. Additionally, the sharp peak at 22.87° transforms into a rounded peak, indicating successful growth of mesoporous SiO_2_ on the surface of SiO_2_@MnO_2_. SiO_2_@MnO_2_@MAF-7 not only displays highly overlapping diffraction peaks at 21.19°, 37.00°, 42.49°, and 56.01° with SiO_2_@MnO_2_, but also presents characteristic diffraction peaks of MAF-7 at 10.4° (200), 12.8° (211), 14.8° (220), and 16.4° (310) [27], demonstrating that the protective shell has successfully encapsulated SiO_2_@MnO_2_.

**Figure 3:**
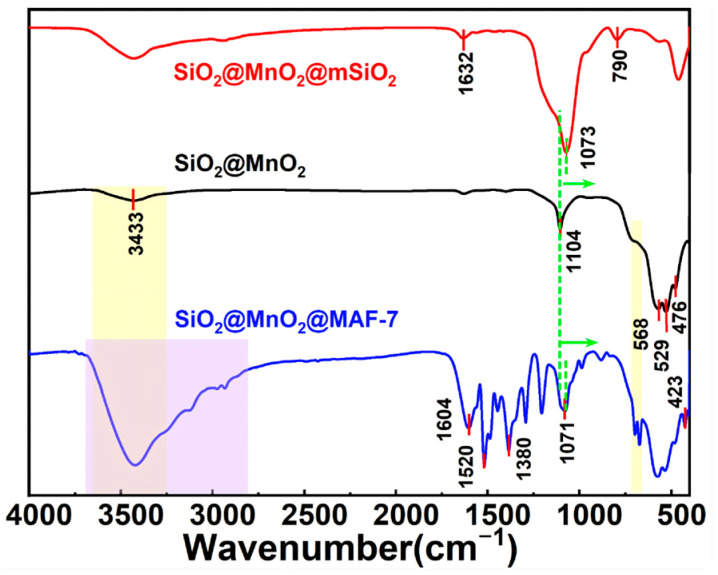
FTIR images of SiO_2_@MnO_2_, SiO_2_@MnO_2_@mSiO_2_, and SiO_2_@MnO_2_@MAF-7.

The FTIR (Figure 3) characteristic peak of SiO_2_@MnO_2_ at 476.58 cm^−1^ is attributed to the bending vibration of the Si-O bond, while the strong and broad absorption band at 1104.57 cm^−1^ corresponds to the antisymmetric stretching vibration of Si-O-Si. The peaks at 529.10 cm^−1^ and 568.48 cm^−1^ represent the symmetrical and antisymmetrical stretching vibrations of Mn-O, respectively, and the peak at 3432.98 cm^−1^ is due to the O-H antisymmetrical stretching vibration. Compared to SiO_2_@MnO_2_, the FTIR characteristic peaks of SiO_2_@MnO_2_@mSiO_2_ show a displacement in the peak position of the antisymmetric stretching vibration of Si-O-Si, an enhancement in the O-H antisymmetric stretching vibration, and the emergence of a new O-H bending vibration at 1632.14 cm^−1^. This indicates the formation of Si-O-Mn covalent bonds between SiO_2_ and MnO_2_. There is an increase in intensity at 1072.5 cm^−1^, and the Si-O symmetrical stretching vibration appears at 790.33 cm^−1^, indicating the successful growth of mesoporous silica on the surface of SiO_2_@MnO_2_. In comparison, the FTIR characteristic peaks of SiO_2_@MnO_2_@MAF-7 at 423.21 cm^−1^ and 1603.92 cm^−1^ are attributed to the Zn-N vibration of MAF-7 and the C=C and C=N bond stretching vibrations of the imidazole ring, respectively, signifying that MAF-7 is successfully wrapped on the surface of SiO_2_@MnO_2_. Peak splitting is observed in the region of 670–700 cm^−1^, with significant broadening of the O-H and N-H stretching vibrations, indicating that SiO_2_@MnO_2_ and MAF-7 are connected by hydrogen bonds. These results demonstrate the successful preparation of the core–shell structure SiO_2_@MnO_2_@MAF-7.

**Figure 4:**
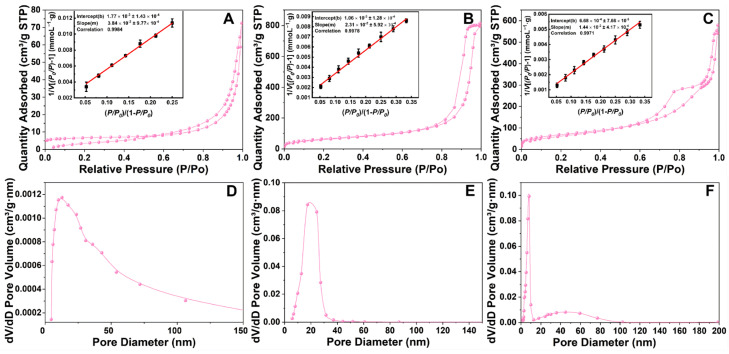
BET images of SiO_2_@MnO_2_, SiO_2_@MnO_2_@mSiO_2_, and SiO_2_@MnO_2_@MAF-7. (**A**–**C**) The adsorption–desorption isotherms and BET linear fitting curves for SiO_2_@MnO_2_, SiO_2_@MnO_2_@mSiO_2_, and SiO_2_@MnO_2_@MAF-7, respectively. (**D**–**F**) The (BJH) pore size distributions of SiO_2_@MnO_2_, SiO_2_@MnO_2_@mSiO_2_, and SiO_2_@MnO_2_@MAF-7.

Figure 4 illustrates the BET data for three materials: SiO_2_@MnO_2_, SiO_2_@MnO_2_@mSiO_2_, and SiO_2_@MnO_2_@MAF-7. Figure 4A,D reveal that the materials possess irregular pore structures formed by the stacking of flake-like particles, indicating the successful preparation of SiO_2_@MnO_2_. Figure 4B displays a typical type IV isotherm for highly ordered mesoporous materials, featuring a distinct H1 hysteresis loop. The specific surface area of SiO_2_@MnO_2_@mSiO_2_ is approximately 238.89 m^2^/g, with an average mesopore diameter of about 17.67 nm (Figure 4E). Figure 4C shows an inconspicuous H1-type hysteresis loop. SiO_2_@MnO_2_@MAF-7 has a specific surface area of 251.99 m^2^/g and an average mesopore diameter of approximately 12.47 nm (Figure 4F). The literature [28] indicates that the size of HRP is 4.0 × 4.4 × 6.8 nm, which corresponds to the mesopore diameter of SiO_2_@MnO_2_@MAF-7.

To validate the reliability of BET analysis, we evaluated N_2_ adsorption data through linear fitting (Figure 4A–C). The linear correlation coefficient R^2^ for the BET equation exceeded 0.99 (*p* < 0.001) across all samples, indicating that the assumptions of the BET theory were satisfied within the relative pressure range of P/P_0_ = 0.05–0.35. This high degree of linearity ruled out interference from factors such as capillary condensation in the calculation of specific surface area. The H1-type hysteresis loop in Figure 4C becomes less pronounced due to partial capillary condensation in MAF-7’s mesopores (12.47 nm), consistent with IUPAC Type IV isotherms for materials with ink-bottle pores [28]. This does not contradict BET validity as confirmed by linearity verification.

### 3.2. Kinetic Studies of Free and Immobilized HRP Using H_2_O_2_ and TMB as Substrates

**Figure 5:**
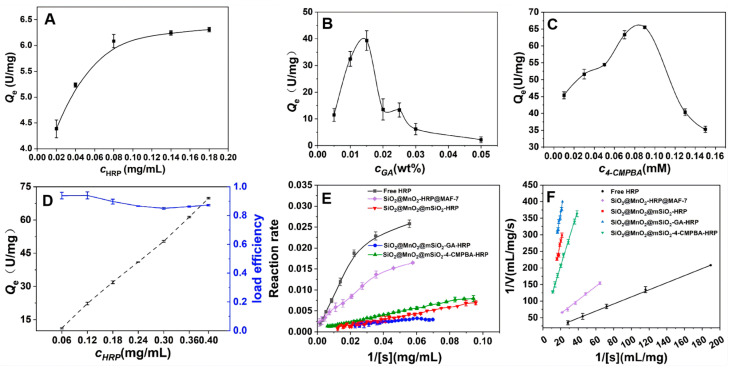
HRP loading capacity and kinetic images of different loading methods. (**A**–**D**) Loading capacities of SiO_2_@MnO_2_@mSiO_2_-HRP, SiO_2_@MnO_2_@mSiO_2_-GA-HRP, SiO_2_@MnO_2_@mSiO_2_-4-CMPBA-HRP, and SiO_2_@MnO_2_-HRP@ MAF-7; the Michaelis–Menten plot (**E**); the Lineweaver–Burk plot (**F**).

The effect of different immobilization methods on the amount of HRP immobilized (Figure 1), and the kinetic results of immobilized HRP are shown in Figure 5. When SiO_2_@MnO_2_@mSiO_2_ directly adsorbs HRP (SiO_2_@MnO_2_@mSiO_2_-HRP, Figure 5A), the amount of immobilization increases with the increase in HRP concentration, reaching a maximum immobilization of 6.09 U/mg at an HRP concentration of 0.08 mg/mL. The immobilization amount obtained by covalently immobilizing HRP using GA as a cross-linking agent (SiO_2_@MnO_2_@mSiO_2_-GA-HRP) is illustrated in Figure 5B, where the immobilization amount first increases and then decreases with increasing HRP concentration. This is attributed to the possibility that at high HRP concentrations, the enzyme molecules already adsorbed on the carrier surface may cross-link with new enzyme molecules via GA, forming a multilayer covering, which leads to the blockage of the active sites and the pores of the carrier. The maximum immobilization amount for SiO_2_@MnO_2_@mSiO_2_-GA-HRP is 39.32 U/mg. The immobilization of HRP using 4-CMPBA as a cross-linking agent (SiO_2_@MnO_2_@mSiO_2_-4-CMPBA-HRP, Figure 5C) shows an initial increase and subsequent decrease in immobilization amount with increasing 4-CMPBA concentration, which is due to excessive modification of the carrier surface by 4-CMPBA at high concentrations, leading to pore blockage. Furthermore, excess 4-CMPBA may also induce conformational changes in the enzyme; the maximum immobilization amount for SiO_2_@MnO_2_@mSiO_2_-4-CMPBA-HRP is 65.52 U/mg. When HRP is directly encapsulated using MAF-7 (SiO_2_@MnO_2_-HRP@MAF-7, Figure 5D), the loading capacity increases with HRP concentration, while the loading efficiency decreases with increasing HRP concentration, and the extent of this decrease becomes smaller. This is attributed to the porous structure of MAF-7 allowing for high loading, with the loading capacity only limited by the pore volume of MAF-7 and the diffusion efficiency of HRP. At low HRP concentrations, the diffusion efficiency is high, and the loading efficiency approaches its peak; at high HRP concentrations, the pores gradually saturate, resulting in diminishing marginal efficiency. The loading efficiency reaches a plateau at an HRP concentration of 0.293 mg/mL, with a loading efficiency of 85.03% and a loading capacity of 50.37 U/mg. The immobilization amounts, in descending order, are as follows: SiO_2_@MnO_2_@mSiO_2_-4-CMPBA-HRP, SiO_2_@MnO_2_-HRP@MAF-7, SiO_2_@MnO_2_@mSiO_2_-GA-HRP, and SiO_2_@MnO_2_@mSiO_2_-HRP.

The reaction rate of enzyme-catalyzed processes increases with the concentration of substrates, but the rate of increase gradually slows down, ultimately converging to a stable value (Figure 5E). From the above results (Figure 5F, Table 1), SiO_2_@MnO_2_@MAF-7 shows a higher affinity for substrates when immobilizing HRP, which is attributed to the microporous and some mesoporous channels of SiO_2_@MnO_2_@MAF-7 that facilitate substrate diffusion and increase the effective contact area between HRP and the substrate. In summary, SiO_2_@MnO_2_@MAF-7 is the optimal carrier for immobilizing HRP.

### 3.3. Characterization of SiO_2_@MnO_2_-HRP@MAF-7

**Figure 6:**
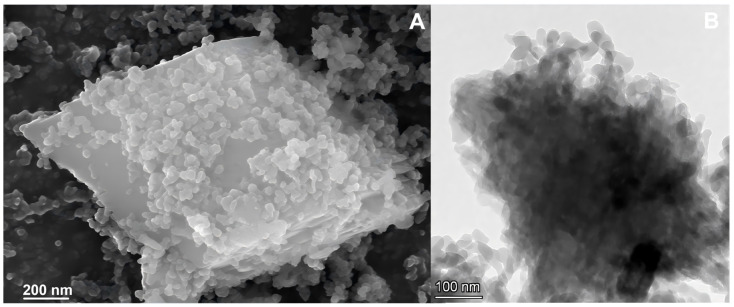
SEM image (**A**) and TEM image (**B**) of SiO_2_@MnO_2_-HRP@MAF-7.

**Figure 7:**
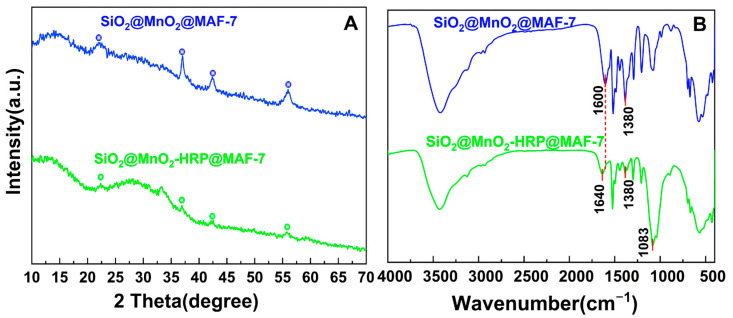
XRD images (**A**) and FTIR images (**B**) of SiO_2_@MnO_2_@MAF-7 and SiO_2_@MnO_2_-HRP@MAF-7.

The SEM and TEM images of SiO_2_@MnO_2_-HRP@MAF-7 are shown in Figure 6. The surface of SiO_2_@MnO_2_-HRP@MAF-7 changes from a rough sponge-like spherical shape (Figure 1D) to a smooth polyhedral shape (Figure 6A). There are no significant changes in the morphology of SiO_2_@MnO_2_@MAF-7 before and after the immobilization of HRP, indicating that the immobilization process does not affect the morphology of the material. From the TEM image (Figure 6B), it can be observed that a large number of MAF-7 particles are distributed on the surface of SiO_2_@MnO_2_-HRP@MAF-7. These changes are related to the immobilization of HRP, as HRP contains nitrogen elements in its peptide bonds, side chains, and some free amino groups, which facilitate its immobilization. The XRD peaks of SiO_2_@MnO_2_-HRP@MAF-7 (Figure 7A) essentially overlap with those of SiO_2_@MnO_2_@MAF-7, with identical diffraction peaks appearing at 12.66°, 22.29°, 36.81°, 42.37°, and 55.95°, indicating that there is no significant difference in the crystal structure of the two. The enzyme immobilized on the carrier SiO_2_@MnO_2_@MAF-7 does not affect its structural integrity or crystal morphology.

As shown in Figure 7, a new peak appears at 1083 cm^−1^ compared to the FTIR image of SiO_2_@MnO_2_@MAF-7, which is attributed to the characteristic vibrations of the HRP porphyrin ring; the peak at 1640 cm^−1^ corresponds to the characteristic Amide I band of HRP. The stretching vibration of the C=N bond in MAF-7 (1600 cm^−1^) is significantly reduced, which is due to the π-π interaction between the aromatic residues of HRP and the imidazole ring of MAF-7 or the formation of hydrogen bonds between the two, weakening the vibrational intensity of the C=N bond. The symmetric bending vibration of the methyl group (-CH_3_) in the MAF-7 ligand (1380 cm^−1^) is also noticeably diminished, attributed to the occupation of MAF-7’s pore channels by HRP, which restricts the internal space of the channels and inhibits the free vibrations of the methyl group (-CH_3_), leading to a decrease in peak intensity. The above results indicate that HRP and MAF-7 are tightly bound through π-π interactions and hydrogen bonding. Furthermore, no shift in XRD peak positions was observed before and after HRP immobilization (Figure 7A), and no new interference peaks appeared, indicating that the chemical reaction did not disrupt the crystal framework. Simultaneously, the retention of characteristic Si-O, Mn-O, and Zn-N peaks in FTIR (Figure 7B) further confirms the chemical stability of immobilized HRP.

**Figure 8:**
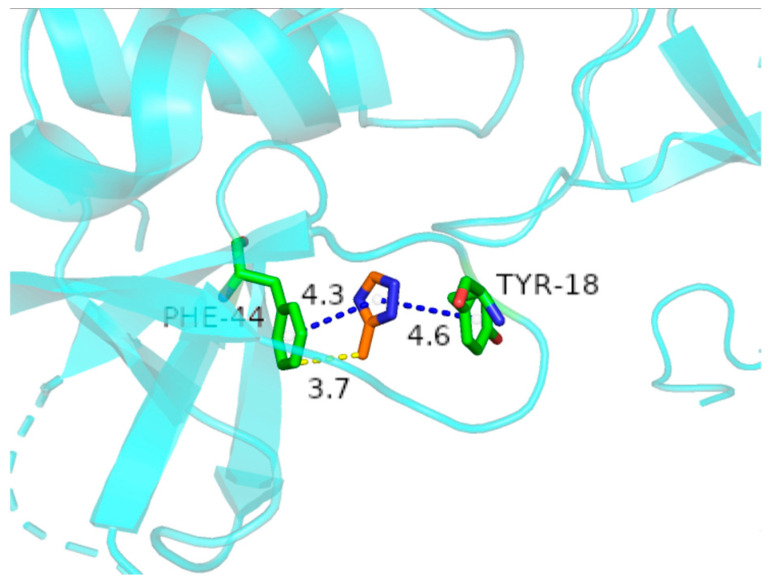
The binding mode of HRP with 3-methyl-1,2,4-triazole. The blue dashed line represents π-π interactions, while the yellow dashed line indicates hydrogen bonds.

The binding poses and interactions of HRP with SiO_2_@MnO_2_@MAF-7 were obtained using Autodock Vina v.1.2.2 (Figure 8). The results indicate that the phenylalanine residues of HRP interact with 3-methyl-1,2,4-triazole through hydrogen bonds and π-π conjugation, while the tyrosine residues of HRP interact with 3-methyl-1,2,4-triazole through π-π conjugation. There exist multiple pairs of phenylalanine and tyrosine positioned relatively as shown in Figure 8, which are closely bound to SiO_2_@MnO_2_@MAF-7 through the aforementioned interactions. The mutual validation through infrared spectroscopy and molecular docking demonstrates that HRP is immobilized on the carrier through π-π and hydrogen bond interactions with MAF-7.

**Figure 9:**
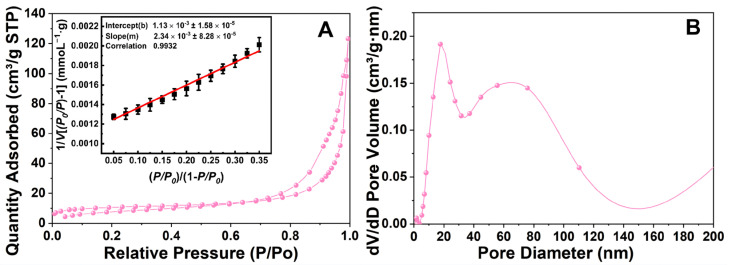
Adsorption–desorption isotherms and BET linear fitting (**A**) and pore size distribution (BJH) of SiO_2_@MnO_2_-HRP@MAF-7 (**B**). Prior to BET measurements, samples were degassed at 120 °C under vacuum (<10^−3^ Torr) for 12 h. FTIR/XRD confirmed no structural degradation (Figure 7), and enzymatic assays verified retained activity (Figure 10), suggesting that HRP’s covalent/confined immobilization prevents thermal denaturation during brief degassing. The appearance of pores > 40 nm (Figure 9B) arises from interparticle voids formed by MAF-7 crystal stacking after HRP loading, as evidenced by SEM (Figure 6A). These macropores enhance substrate diffusion without compromising enzyme stability.

After the fixation of HRP, the specific surface area decreased to 39.40 m^2^/g, and the pore volume changed from 0.89 cm^3^/g to 0.32 cm^3^/g. This may be due to HRP molecules being immobilized within the pores of MAF-7, blocking some of its pore channels. The pore diameter distribution increased from 12.47 nm to 24.58 nm, indicating that HRP selectively occupies mesopores [29]. Figure 9 shows that SiO_2_@MnO_2_@MAF-7, after immobilizing HRP, exhibits a significant H3-type hysteresis loop, indicating a transition in the material’s pore structure from uniform mesopores to open layered/slit pores. The BJH pore size distribution demonstrates a significant reduction in the proportion of pores < 10 nm, with the emergence of a new peak in the region > 40 nm, indicating that the stacking of materials has formed larger open pores. In summary, the hysteresis loop type shifted from H1 to H3 after HRP immobilization (Figure 9A), which can be attributed to the following: (1) partial mesopore blockage caused by HRP loading within MAF-7 channels; (2) enzyme molecules inducing altered MAF-7 crystal stacking patterns, forming larger interlayer/slit pores. This phenomenon aligns with previous reports on pore structure transformations in MOFs induced by enzyme immobilization [28,29].

**Figure 10:**
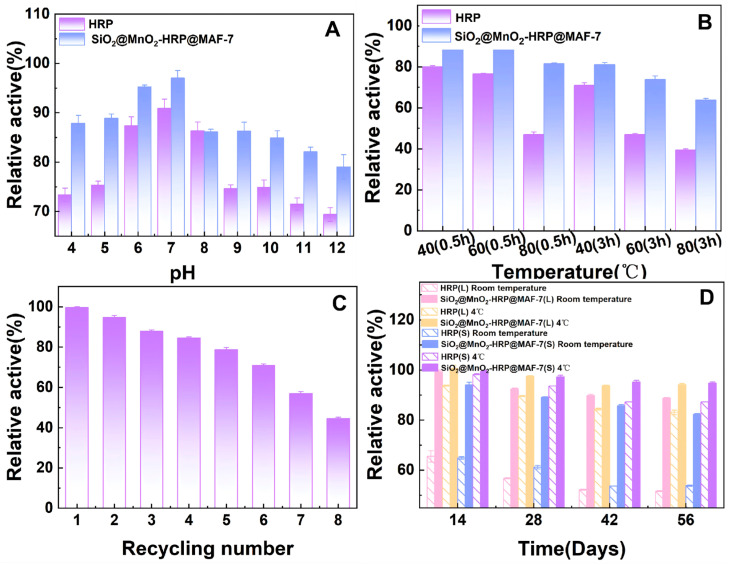
pH stability (**A**), thermal stability (**B**), reusability (**C**), and storage stability (**D**) of free and immobilized HRP.

### 3.4. Determination of Enzyme Properties

The aforementioned characterization experiments successfully demonstrated that SiO_2_@MnO_2_@MAF-7 can be used as a carrier material for immobilized HRP. We investigated the acid-base stability, thermal stability, storage stability, and reusability of SiO_2_@MnO_2_-HRP@MAF-7, as shown in Figure 10.

#### 3.4.1. The Optimal pH for Free HRP and SiO_2_@MnO_2_-HRP@MAF-7

The activity of free enzymes is highly susceptible to changes in pH. The relative activity variations in HRP before and after immobilization were studied at different pH levels. As shown in Figure 10A, after incubating for 2.5 h under the same pH conditions, SiO_2_@MnO_2_-HRP@MAF-7 exhibited higher activity than free HRP. Both SiO_2_@MnO_2_-HRP@MAF-7 and free HRP had an optimum pH of 7, with free HRP retaining 90.90% of its relative activity, while SiO_2_@MnO_2_-HRP@MAF-7 retained 97.00% of its relative activity. As pH increases or decreases, enzyme activity diminishes. When the pH reaches 9, the relative activity of free HRP is only 74.65%, whereas immobilized HRP retains 86.25% of its enzyme activity. Similarly, at pH = 4, the activity of free enzymes is approximately 73.35%, while the activity of the immobilized enzymes is retained at 87.88%.

In summary, immobilized enzymes exhibit relatively stable activity in extreme pH environments, attributed to the enzymes being anchored to a support, thereby reducing enzyme denaturation or desorption caused by pH changes, and lowering the risk of deactivation induced by conformational changes [30,31]. Additionally, the SiO_2_@MnO_2_@MAF-7 multilayer core–shell structure forms a physical and chemical buffering barrier, which can mitigate the impact of drastic external pH fluctuations on horseradish peroxidase (HRP) [32]. The synergistic effect of the multi-material components creates a broad pH tolerance window, which can reduce environmental aggressiveness. Therefore, the immobilization of HRP on SiO_2_@MnO_2_@MAF-7 has been shown to enhance the enzyme’s pH stability to a certain extent.

#### 3.4.2. The Optimal Temperature for the Free HRP and SiO_2_@MnO_2_-HRP@MAF-7

The activity of both free and immobilized HRP decreases with increasing temperature and extended incubation time, as shown in Figure 10B. Under the same conditions, the immobilized enzyme exhibits a more stable relative activity; after incubation at 80 °C for 0.5 h, the activity of the immobilized enzyme is maintained at 81.45%, while that of the free enzyme is only 46.91%. This demonstrates that SiO_2_@MnO_2_@MAF-7 provides a more stable protective framework for the enzyme, thereby reducing the impact of the thermal environment on the enzyme [33,34].

#### 3.4.3. The Reusability of Free HRP and SiO_2_@MnO_2_-HRP@MAF-7

Free enzymes are often difficult to recover when used, making reuse impossible. The primary purpose of enzyme immobilization is to enhance the reusability of enzymes. The results of the reusability of SiO_2_@MnO_2_-HRP@MAF-7 are shown in Figure 10C. After five cycles, the carrier retained 65% of its activity, indicating structural integrity, as severe degradation would cause a sharp decline in activity (e.g., >50% reduction). Furthermore, no visible precipitation or color change was observed during centrifugation, further confirming that the carrier remained intact. The enzyme activity of the immobilized enzyme decreases sequentially with an increase in the number of cycles, which is mainly attributed to the loss of carrier quality, leaching of non-covalently bound enzymes after repeated washing, and the oxidation of the key active site amino acids of the enzyme due to the Fenton reaction in the presence of H_2_O_2_ [35]. Calculations indicate that the first cycle exhibited a leakage rate of approximately 5.8%, while cycles 2–5 showed an average leakage rate of 1.7% each, totaling 12.4%. This trend does not fully correlate with the observed activity decline (35%), further suggesting that the primary cause of activity loss stems from enzyme conformational changes rather than desorption.

#### 3.4.4. The Storage Stability of Free HRP and SiO_2_@MnO_2_-HRP@MAF-7

The results of storage stability are shown in Figure 10D. Regardless of the storage method, the activity of immobilized enzymes is consistently much higher than that of free enzymes. This may be due to the strong π-π conjugation and hydrogen bond interactions formed between HRP and the carrier, which make the spatial structure of the enzyme less susceptible to damage. Furthermore, the synergistic effect of the carrier provides a protective framework for the enzyme. Under room temperature conditions, the relative activity of the enzyme solution is always slightly higher than that of the solid powder enzyme, possibly because the aggregation of the powder leads to masking of the active sites [36], while the enzyme solution benefits from hydration protection. During the first 28 days of storage at 4 °C, the relative activity of the enzyme solution remains slightly higher than that of the solid powder enzyme. However, between 28 and 56 days, both exhibit comparable relative activities. The initially higher enzyme activity in the solution is mainly due to losses during the freeze-drying rehydration process, while the later convergence is attributed to the dual effects of hydrolysis accumulation in the solution and the protection provided by the solid glassy state.

## 4. Comparison with Other Hybrid Carriers

As shown in Table 2, the SiO_2_@MnO_2_@MAF-7 hybrid carrier developed in this study demonstrates significant advantages over other recently reported hybrid carrier systems in terms of HRP immobilization performance. Specifically, this carrier system exhibits outstanding enzyme loading capacity (50.37 U/mg) and shows significant improvements in key metrics such as operational stability (including thermal stability and pH tolerance) and reusability compared to typical carriers like Fe_3_O_4_@ZIF-8 [15] and MIL-100(Fe) [28]. It demonstrates significant improvements in key metrics such as operational stability (including thermal stability and pH tolerance) and reusability. This performance advantage stems from its unique three-dimensional hierarchical structure design: (1) the core SiO_2_ nanoparticles confer excellent mechanical strength to the carrier; (2) the intermediate MnO_2_ layer optimizes enzyme orientation through metal coordination and generates significant catalytic synergistic effects; (3) the outer MAF-7 framework not only provides high-density enzyme immobilization sites but also effectively protects the enzyme active sites through spatial confinement due to its regular mesoporous structure. Based on these characteristics, this composite carrier demonstrates significant application potential across multiple fields, including the enzyme-catalyzed degradation of phenolic pollutants in industrial wastewater treatment, the construction of highly stable electrochemical biosensors, the development of enzyme-linked immunosorbent assay platforms in diagnostic reagents, and the preparation of highly efficient biocatalysts for fine chemical synthesis.

## 5. Conclusions

This study first designed and successfully constructed a SiO_2_@MnO_2_@MAF-7 composite carrier with a hierarchical porous structure and catalytic synergistic function, systematically comparing the effects of adsorption, cross-linking, and embedding methods on the immobilization of Horseradish Peroxidase (HRP). The experimental results indicate that the SiO_2_@MnO_2_-HRP@MAF-7 obtained through the embedding method exhibited the optimal enzyme immobilization performance, with a loading capacity of 50.37 U/mg (loading efficiency of 85.03%), and a significantly lower Km value compared to free HRP, confirming that the carrier enhances substrate affinity through the synergistic interaction of multi-level pore channels and Mn^2+^ coordination. Compared to free enzymes, the introduction of the SiO_2_@MnO_2_@MAF-7 carrier not only increased the loading capacity but also significantly improved HRP’s acid–base resistance, thermal stability, storage stability, and reusability. This study not only provides a high-performance immobilization scheme for the industrial application of HRP but also suggests that the design strategy of a rigid core-functional shell-porous framework can be extended to the immobilization research of other redox enzymes, with future optimizations in recovery efficiency through surface modifications of the carrier. The SiO_2_@MnO_2_@MAF-7 carrier exhibits three key competitive advantages over existing hybrid supports: (1) The synergistic ‘rigid core-catalytic shell-porous framework’ design enables simultaneously high loading capacity (50.37 U/mg) and stability (65% activity retention after 5 cycles); (2) Mn^2+^/Zn^2+^ dual-metal coordination optimizes enzyme orientation, reducing Km by 42%; (3) MAF-7’s mesopores (12.47 nm) provide size-matching confinement that minimizes leaching while maintaining substrate accessibility.

## Figures and Tables

**Table 1:** Comparison of kinetic constants for different solid loading methods.

Fixed Mounting Method	Km (mL/mg)	Vmax (mL/mg/s)
HRP	0.1769	0.1653
SiO_2_@MnO_2_@mSiO_2_-HRP	0.5038	0.0398
SiO_2_@MnO_2_@mSiO_2_-GA-HRP	0.3659	0.0245
SiO_2_@MnO_2_@mSiO_2_-4-CMPBA-HRP	0.2812	0.0325
SiO_2_@MnO_2_-HRP@MAF-7	0.0995	0.0481

**Table 2:** Performance comparison of SiO_2_@MnO_2_@MAF-7 with similar supports.

Carrier Type	Load Capacity (U/mg)	Thermal Stability (80 °C)	Reusability	References
SiO_2_@MnO_2_@MAF-7 (This Study)	50.37	81.45% (0.5 h)	65% (5 Times)	--
Fe_3_O_4_@ZIF-8	38.2	60% (0.5 h)	50% (5 Times)	[15]
MIL-100(Fe)	42.1	75% (0.5 h)	58% (5 Times)	[28]

## Data Availability

The original contributions presented in this study are included in the article. Further inquiries can be directed to the corresponding authors.
